# Association Between Thyroid Hormone Imbalance and Cardiovascular Health: An Institution-Based Cross-Sectional Study of Outpatients and Community Screening Participants

**DOI:** 10.7759/cureus.111119

**Published:** 2026-06-18

**Authors:** Wardah Ikram

**Affiliations:** 1 Endocrinology and Diabetes, Allama Iqbal Medical College, Lahore, PAK

**Keywords:** cardiovascular health, ecg abnormalities, hypertension, hyperthyroidism, hypothyroidism, population-based study, thyroid dysfunction

## Abstract

Background and objective

Thyroid hormone imbalance has important cardiovascular implications, influencing cardiac rhythm, blood pressure regulation, and vascular function. The objective of this study was to evaluate the association between thyroid hormone imbalance and cardiovascular abnormalities, including hypertension, ECG abnormalities, arrhythmias, and dyslipidemia, among adults in an institution-based cross-sectional setting.

Methods

This institution-based cross-sectional study was conducted at Allama Iqbal Medical College, Lahore, from January 1, 2019, to December 31, 2019. A total of 424 participants were recruited using nonprobability consecutive sampling. Based on thyroid function tests (triiodothyronine, thyroxine, and thyroid-stimulating hormone), participants were categorized into euthyroid, hypothyroid, and hyperthyroid groups. Cardiovascular assessment included blood pressure measurement and resting ECG. Data were analyzed using IBM SPSS Statistics for Windows, version 25.0 (released 2017; IBM Corp., Armonk, NY, USA). The chi-square test was applied for categorical variables, and a p-value < 0.05 was considered statistically significant. Multivariable logistic regression was also performed to adjust for potential confounders.

Results

Among the 424 participants, 238 (56.13%) were euthyroid, 126 (29.72%) were hypothyroid, and 60 (14.15%) were hyperthyroid. The prevalence of hypertension and ECG abnormalities was higher in the hypothyroid and hyperthyroid groups than in euthyroid individuals. ECG abnormalities were observed in 28 (11.76%) euthyroid, 48 (38.10%) hypothyroid, and 36 (60.00%) hyperthyroid participants. Overall cardiovascular manifestations, including tachycardia, bradycardia, atrial fibrillation, ischemic changes, and left ventricular strain, were more frequent in the thyroid dysfunction groups. Cardiovascular risk factors such as hypertension, dyslipidemia, and arrhythmias showed a significant association with thyroid dysfunction (p < 0.001).

Conclusions

Thyroid hormone imbalance is significantly associated with cardiovascular abnormalities in the studied population. These findings highlight the importance of early detection and cardiovascular risk assessment in individuals with thyroid dysfunction.

## Introduction

Thyroid hormones are essential for the control of several physiological processes, such as metabolism, growth, thermogenesis, and cardiovascular function [1,2]. The direct and indirect effects of triiodothyronine (T3) and thyroxine (T4) on the heart and vascular system include effects on myocardial contractility, heart rate, systemic vascular resistance, and lipid metabolism [3]. Thyroid disorders are common worldwide, with an estimated prevalence of up to 10-15% among adults, and many cases remain undiagnosed [4]. Clinically significant cardiovascular changes may occur even with subtle alterations in thyroid hormone levels, underscoring the relationship between endocrine and cardiovascular function [5].

Thyroid hormone disorders, including hypothyroidism and hyperthyroidism, are associated with a variety of cardiovascular manifestations [6]. Bradycardia, diastolic systemic hypertension, increased systemic vascular resistance, dyslipidemia, and accelerated atherosclerosis are commonly associated with hypothyroidism. These changes increase the risk of coronary artery disease and heart failure [7]. By contrast, hyperthyroidism is associated with tachycardia, elevated cardiac output, atrial fibrillation, and widened pulse pressure, which can predispose individuals to arrhythmias and high-output cardiac failure [8]. In addition, long-term cardiovascular alterations may occur in subclinical forms of thyroid dysfunction, which often remain undiagnosed [9].

Myocardial and vascular tissues possess thyroid hormone receptors and are especially sensitive to variations in thyroid hormone levels [10]. These hormones regulate the expression of myocardial contractile proteins, the sensitivity of beta-adrenergic receptors, and calcium handling in cardiac cells [11]. Thyroid dysfunction also affects lipid profiles, endothelial function, and inflammatory pathways, further contributing to cardiovascular risk [12]. Numerous studies conducted in different populations have demonstrated associations between thyroid dysfunction and hypertension, arrhythmias, and ischemic heart disease; however, the prevalence and severity of these conditions vary by region. The increasing prevalence of thyroid disorders and their association with cardiovascular disease represent significant clinical concerns, particularly in populations where routine screening is not feasible [13,14].

Understanding the cardiovascular implications of thyroid dysfunction is complicated by variations in thyroid status across different age groups, sexes, and metabolic conditions. Population-based assessments are therefore essential for providing a comprehensive understanding of thyroid-related cardiovascular abnormalities beyond hospital-based observations. Such studies can help identify the burden of both overt and subclinical thyroid dysfunction and their associated cardiovascular manifestations within the general population. Given the limited population-based evidence from Pakistan, further investigation is warranted to better characterize these associations and inform early screening and preventive strategies. This study was conducted to evaluate the association between thyroid hormone imbalance and cardiovascular abnormalities, including hypertension, ECG abnormalities, arrhythmias, and dyslipidemia, in a population-based setting.

## Materials and methods

Study design and setting

This cross-sectional institution-based observational study was conducted at Allama Iqbal Medical College (AIMC), Lahore. Participants from OPDs and community-based screening centers affiliated with the institute were included to ensure general population participation.

Study duration

This study was conducted over 12 months, from January 1, 2019, to December 31, 2019.

Inclusion and exclusion criteria

All men and women aged 18 years or older who agreed to participate were included in the study. Participants were required to undergo thyroid function testing and cardiovascular evaluation. Patients with known congenital heart disease, chronic renal failure, chronic liver disease, or pregnancy were excluded because these physiological or pathological conditions could affect the interpretation of thyroid or cardiovascular parameters. Patients receiving medications known to affect thyroid function, including amiodarone, lithium, and long-term corticosteroids, as well as critically ill patients and those unwilling to participate, were also excluded.

Sample size and sampling technique

A total of 424 participants were included in the study. The sample size was calculated using the standard formula for estimating a population proportion:

$n = \frac{Z^{2} \, p (1-p)}{d^{2}},$

where n = required sample size, Z = Z value for a 95% confidence level (1.96), p = expected prevalence of thyroid dysfunction in the population, and d = margin of error (0.05) [15].

The calculation was based on the expected prevalence of thyroid dysfunction in the general population, a 95% confidence level, and a 5% margin of error to ensure adequate statistical power. After applying the formula, the required minimum sample size was achieved and slightly exceeded to improve precision. A nonprobability consecutive sampling technique was used, and participants were recruited until the required sample size was met.

Data collection procedure

A structured pro forma was developed by the research team in consultation with clinical experts to ensure the content validity, clarity, and completeness of the data collection process (Appendix A). All participants provided informed consent, and demographic information, cardiac risk factors, and relevant medical history were obtained. To ensure uniformity in data collection, all participants underwent cardiovascular assessment and thyroid function testing under standard conditions.

Clinical and biochemical assessment

Venous blood samples collected under aseptic conditions were used to perform thyroid function tests using standardized immunoassay techniques, including serum T3, T4, and thyroid-stimulating hormone (TSH). Internal laboratory quality control procedures were followed to ensure assay reliability and consistency. Participants were divided into three groups: (1) euthyroid; (2) hypothyroid; and (3) hyperthyroid, based on the reference ranges of TSH (0.4-4.0 mIU/L), T3 (0.8-2.0 ng/mL), and T4 (5-12 µg/dL).

Cardiovascular evaluation consisted of blood pressure measurement using a calibrated mercury sphygmomanometer (or validated aneroid device) under standard resting conditions, with appropriate cuff size selection and duplicate readings recorded to minimize measurement error. A resting 12-lead ECG was performed to assess cardiac rhythm, ischemic changes, and conduction abnormalities.

ECG abnormalities were defined according to standard clinical criteria and included arrhythmias (atrial fibrillation, premature ventricular contractions, tachycardia >100 beats/min, and bradycardia <60 beats/min), ischemic changes (ST-segment elevation or depression and T-wave inversion), and conduction defects (atrioventricular block and bundle branch block patterns). All ECGs were independently interpreted by two cardiologists blinded to the biochemical results, and discrepancies were resolved by consensus. Additional cardiovascular findings, such as clinical symptoms or examination-based abnormalities, were documented when clinically indicated.

Variables of the study

Thyroid hormone levels (T3, T4, and TSH) and thyroid functional status (euthyroid, hypothyroid, and hyperthyroid) were used as independent variables. Cardiovascular variables included systolic and diastolic blood pressure, ECG-defined abnormalities, and other cardiovascular manifestations, such as arrhythmias, tachycardia, and bradycardia. Potential confounding variables, including age, sex, BMI, smoking status, diabetes mellitus, hypertension, and lipid profile, were considered in the analysis. These variables were controlled for using stratification and multivariable statistical analysis, where appropriate, to minimize confounding bias.

Statistical analysis

Data were analyzed using IBM SPSS Statistics for Windows, version 25.0 (released 2017; IBM Corp., Armonk, NY, USA). Quantitative variables were expressed as mean ± SD, whereas qualitative variables were presented as frequencies and percentages. The normality of continuous variables was assessed before inferential analysis.

For bivariate analysis, categorical variables were compared using the chi-square test, whereas continuous variables were analyzed using the independent t-test or one-way ANOVA, as appropriate. To control for potential confounding, multivariable logistic regression analysis was performed, adjusting for key covariates, including age, sex, and BMI. Adjusted ORs with 95% CIs were reported to determine the independent association between thyroid dysfunction and cardiovascular outcomes. A p-value < 0.05 was considered statistically significant.

Ethical considerations

The study was approved by the Institutional Review Board of AIMC, Lahore, before the study commenced. All participants provided written informed consent after being informed of the objectives and procedures of the study. Participant data were kept confidential, and anonymity was maintained throughout the study. Participants were informed that they could withdraw from the study at any time without affecting their medical treatment.

## Results

A total of 424 participants were included in the study (Table 1). The age distribution showed that 102 (24.06%) participants were aged 18-30 years, 148 (34.91%) were aged 31-45 years, 114 (26.89%) were aged 46-60 years, and 60 (14.15%) were older than 60 years. There were slightly more females (228 (53.77%)) than males (196 (46.23%)). Regarding BMI status, 162 (38.21%) participants had a normal BMI, 176 (41.51%) were overweight, and 86 (20.28%) were obese.

**Table 1:** Baseline demographic characteristics of study participants (n = 424) Data are presented as frequencies (n) and percentages (%). Age, sex, and BMI distributions of participants are described. No inferential statistical tests were applied, as the data are descriptive in nature.

Variable	Category	Number (n)	Percentage (%)
Age (years)	18-30	102	24.06
	31-45	148	34.91
	46-60	114	26.89
	>60	60	14.15
Gender	Male	196	46.23
	Female	228	53.77
BMI status	Normal	162	38.21
	Overweight	176	41.51
	Obese	86	20.28

**Figure 1:**
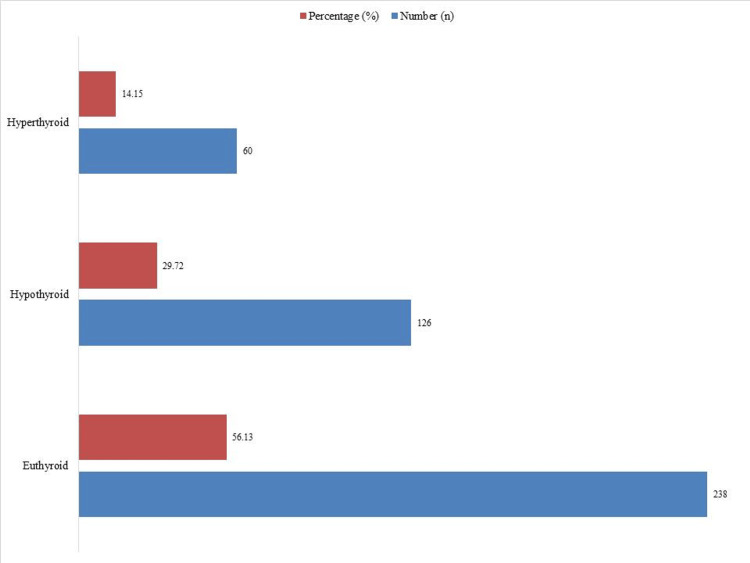
Distribution of thyroid status among participants (n = 424) Data are presented as frequencies (n) and percentages (%). Thyroid status was categorized as euthyroid, hypothyroid, and hyperthyroid based on serum thyroid function tests. No statistical test was applied because the figure represents a descriptive distribution.

Among the 424 participants, 238 (56.13%) were euthyroid, 126 (29.72%) were hypothyroid, and 60 (14.15%) were hyperthyroid, indicating that approximately one-third of the population had thyroid dysfunction (Figure 1).

A statistically significant association was observed between thyroid functional status and blood pressure categories (Table 2). The prevalence of hypertension increased progressively from the euthyroid to the hypothyroid groups and was highest among hyperthyroid participants, indicating a clear trend toward worsening blood pressure profiles with thyroid dysfunction. These findings suggest that both hypothyroid and hyperthyroid states are associated with an increased risk of hypertension compared with the euthyroid state, demonstrating a strong relationship between thyroid imbalance and altered blood pressure regulation.

**Table 2:** Association between thyroid status and blood pressure categories Data are presented as frequencies (n) and percentages (%). A p-value < 0.05 was considered statistically significant.

Thyroid status	Normal blood pressure, n (%)	Hypertension, n (%)	Total
Euthyroid	180 (75.63%)	58 (24.37%)	238
Hypothyroid	62 (49.21%)	64 (50.79%)	126
Hyperthyroid	18 (30.00%)	42 (70.00%)	60
Chi-square test (χ²)	58.62		
p-value	<0.001		

A significant difference in mean systolic and diastolic blood pressure was observed across the thyroid status groups (Table 3). Participants with thyroid dysfunction exhibited higher average blood pressure values than euthyroid individuals, with a stepwise increase from the euthyroid to the hypothyroid and hyperthyroid groups. These results indicate that thyroid dysfunction is associated not only with categorical hypertension but also with a measurable increase in overall blood pressure levels, reflecting its impact on cardiovascular hemodynamics.

**Table 3:** Comparison of mean blood pressure across thyroid status (ANOVA) Data are presented as mean ± SD. Differences among groups were assessed using one-way ANOVA. A p-value < 0.05 was considered statistically significant.

Thyroid status	Systolic blood pressure (mean ± SD)	Diastolic blood pressure (mean ± SD)
Euthyroid	118.6 ± 12.4	76.2 ± 8.9
Hypothyroid	131.8 ± 14.1	83.5 ± 9.6
Hyperthyroid	142.3 ± 15.2	88.7 ± 10.3
F-value	38.72	29.44
p-value	<0.001	<0.001

ECG findings revealed a higher prevalence of abnormalities among the thyroid dysfunction groups (Table 4). A normal ECG was observed in 210 (88.24%) euthyroid individuals, 78 (61.90%) hypothyroid individuals, and 24 (40.00%) hyperthyroid participants, whereas abnormal ECG findings were present in 28 (11.76%), 48 (38.10%), and 36 (60.00%) participants, respectively, demonstrating increasing cardiac electrical disturbances with thyroid dysfunction.

**Table 4:** ECG abnormalities according to thyroid status (n = 424) Data are presented as frequencies (n) and percentages (%).

Thyroid status	Normal ECG	Abnormal ECG	Total
Euthyroid	210 (88.24%)	28 (11.76%)	238
Hypothyroid	78 (61.90%)	48 (38.10%)	126
Hyperthyroid	24 (40.00%)	36 (60.00%)	60

**Figure 2:**
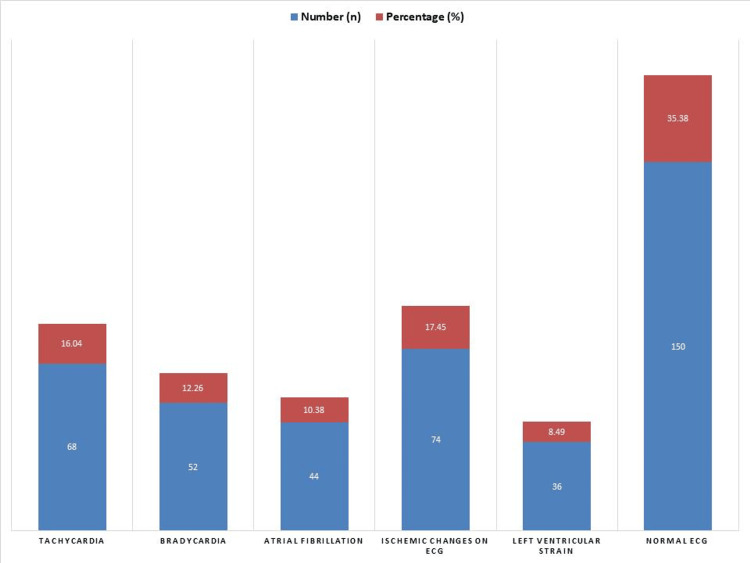
Types of cardiovascular abnormalities in the study population (n = 424) Data are presented as frequencies (n) and percentages (%). The figure shows the distribution of cardiovascular manifestations among participants. No inferential statistical test was applied because the figure is descriptive.

Overall cardiovascular abnormalities included tachycardia in 68 (16.04%) participants, bradycardia in 52 (12.26%), atrial fibrillation in 44 (10.38%), ischemic ECG changes in 74 (17.45%), and left ventricular strain in 36 (8.49%), whereas 150 (35.38%) participants had normal ECG findings, indicating a substantial burden of cardiovascular involvement in the study population (Figure 2).

A strong statistically significant association was observed between thyroid dysfunction and cardiovascular risk factors (Table 5). Hypertension was present in 24.37% of euthyroid, 50.79% of hypothyroid, and 70.00% of hyperthyroid participants (p < 0.001). Dyslipidemia occurred in 27.73%, 57.14%, and 46.67% of participants, respectively, whereas arrhythmias were observed in 11.76%, 38.10%, and 60.00%, respectively. Cardiac symptoms were also significantly more common in the hypothyroid (55.56%) and hyperthyroid (66.67%) groups than in the euthyroid group (22.69%), with all associations reaching statistical significance (p < 0.001).

**Table 5:** Association of thyroid dysfunction with cardiovascular risk factors (crude and adjusted analysis) Data are presented as frequencies (n) and percentages (%). Crude associations were assessed using the chi-square test. Multivariable logistic regression analysis was performed after adjustment for age, sex, and BMI as potential confounding variables. Adjusted ORs with 95% CIs are reported. A p-value < 0.05 was considered statistically significant.

Risk factor	Euthyroid (n = 238)	Hypothyroid (n = 126)	Hyperthyroid (n = 60)	χ²	p-value	Adjusted OR (hypo)	Adjusted OR (hyper)	95% CI	p-value (adjusted)
Hypertension	58 (24.37%)	64 (50.79%)	42 (70.00%)	58.62	<0.001	2.31	3.88	1.45-6.91	<0.001
Dyslipidemia	66 (27.73%)	72 (57.14%)	28 (46.67%)	39.84	<0.001	1.94	2.41	1.22-4.88	0.003
Arrhythmias	28 (11.76%)	48 (38.10%)	36 (60.00%)	74.15	<0.001	2.84	4.92	1.69-8.74	<0.001
Cardiac symptoms	54 (22.69%)	70 (55.56%)	40 (66.67%)	61.28	<0.001	2.12	3.45	1.33-6.22	<0.001

## Discussion

In the present institution-based cross-sectional study, a considerable proportion of participants demonstrated thyroid dysfunction, including hypothyroidism (29.72%) and hyperthyroidism (14.15%), while 56.13% were euthyroid. Overall, approximately 44% of the study population exhibited some degree of thyroid imbalance, indicating a substantial burden of thyroid dysfunction in the sampled population. The reported prevalence of thyroid dysfunction varies widely across different populations and settings, with higher detection rates often observed in screened or clinical cohorts compared with general population estimates. Several studies have also highlighted the presence of undiagnosed or subclinical thyroid abnormalities in apparently asymptomatic individuals, which is consistent with the findings of the present study, suggesting a significant hidden burden of thyroid dysfunction [16].

Cardiovascular changes were observed across thyroid status groups, with a higher prevalence of hypertension in hypothyroid (50.79%) and hyperthyroid (70.00%) participants compared with euthyroid individuals (24.37%). This pattern indicates a significant association between thyroid dysfunction and blood pressure abnormalities. Previous studies have similarly reported that hypothyroidism is more commonly associated with diastolic hypertension, whereas hyperthyroidism is associated with systolic hypertension, which may be explained by alterations in systemic vascular resistance and cardiac output dynamics [17].

Cardiovascular involvement was further reflected by ECG abnormalities, which were more frequent in the hyperthyroid group (60.00%) and hypothyroid group (38.10%) than in the euthyroid group (11.76%). These findings are consistent with existing evidence suggesting that thyroid hormones influence cardiac electrophysiology and may contribute to arrhythmias and conduction disturbances. Prior literature has reported increased risks of atrial fibrillation, tachycardia, and bradycardia in patients with thyroid dysfunction, supporting the observed trend of increasing ECG abnormalities with thyroid imbalance [18].

In the present study, specific cardiovascular manifestations included tachycardia, bradycardia, atrial fibrillation, and ischemic ECG changes. A higher proportion of arrhythmias was observed among hyperthyroid participants, consistent with previous studies reporting increased susceptibility to atrial fibrillation in hyperthyroidism. This is thought to be related to enhanced cardiac excitability and increased sympathetic activity described in the literature [19].

Thyroid dysfunction was significantly associated with cardiovascular risk factors such as hypertension, dyslipidemia, and arrhythmias (p < 0.001). Distinct cardiovascular risk profiles were observed across groups, with hypothyroid participants showing higher dyslipidemia and hyperthyroid participants showing higher arrhythmia prevalence. These findings are consistent with cohort studies indicating that both hyperthyroidism and hypothyroidism are independently associated with increased cardiovascular morbidity through different metabolic and hemodynamic mechanisms [20].

Overall, the present findings demonstrate a progressive increase in cardiovascular risk across thyroid dysfunction groups. This is consistent with existing evidence suggesting that even subclinical thyroid dysfunction may contribute to adverse cardiovascular outcomes. Thyroid hormones play a critical role in regulating cardiac function, vascular tone, and electrophysiological stability, and even minor alterations in hormone levels may result in measurable cardiovascular effects, as observed in this study [21].

Strength and limitations

This study has several strengths. It included a relatively large sample size of 424 participants, enhancing the statistical robustness of the findings. The institution-based cross-sectional design, incorporating both outpatient and community screening participants, improves the external validity of the results compared with single-setting hospital-based studies. A key strength is the simultaneous assessment of biochemical thyroid parameters (T3, T4, and TSH) alongside cardiovascular evaluations, including blood pressure measurements and ECG, enabling a comprehensive evaluation of thyroid-cardiovascular interactions. Standardized laboratory procedures and structured data collection tools further strengthened data reliability and internal validity. In addition, multivariable logistic regression analysis was performed to adjust for key confounders, including age, sex, and BMI, thereby reducing the potential impact of confounding on the observed associations.

Despite these strengths, several limitations should be acknowledged. The use of nonprobability consecutive sampling may introduce selection bias, and although participants were recruited from both OPDs and affiliated community screening centers, the single-institution design may still limit generalizability to the broader population. Furthermore, while multivariable adjustment was performed for key confounders, residual confounding from unmeasured factors such as smoking status, dietary habits, physical activity, and socioeconomic status cannot be excluded. Cardiovascular assessment was limited to blood pressure measurement and resting ECG, without more advanced investigations such as echocardiography or ambulatory ECG monitoring, which restricts detailed structural and functional cardiac evaluation. Finally, the cross-sectional design limits causal inference and does not allow assessment of temporal relationships or long-term cardiovascular outcomes.

## Conclusions

The present study demonstrates a significant association between thyroid hormone imbalance and cardiovascular abnormalities in the study population. The prevalence of hypertension, ECG changes, and other cardiac abnormalities was higher in both hypothyroid and hyperthyroid groups compared with euthyroid individuals, with a progressive increase observed across categories of thyroid dysfunction. These findings indicate that thyroid imbalance is associated with measurable cardiovascular alterations, even in individuals who may be asymptomatic. The results highlight the potential importance of early identification and monitoring of thyroid dysfunction in both clinical and community settings to support timely cardiovascular risk assessment and management. Future studies using longitudinal designs with broader cardiovascular profiling are recommended to better clarify temporal relationships between thyroid dysfunction and cardiac risk. Clinically, routine thyroid screening and early cardiovascular risk assessment may help in timely identification and management of at-risk individuals.

## Appendices

Appendix A: Study questionnaire 

**Table 6:** Structured study questionnaire

Section	Variable/item	Response format/options	Details/coding
A. Participant identification	Study ID	Numeric code	
	Date of assessment	DD/MM/YYYY	
	Study site	AIMC/OPD/Community center	
B. Demographic data	Age	Years (numeric)	
	Gender	Male/Female	
	Marital status	Single/Married/Other	
	Residence	Urban/Rural	
C. Anthropometric data	Height	cm	
	Weight	kg	
	BMI	kg/m²	
D. Lifestyle factors (confounders)	Smoking status	Never/Former/Current	
	Physical activity	Sedentary/Moderate/Active	
	Alcohol use	Yes/No	
E. Medical history (confounders)	Hypertension	Yes/No	
	Diabetes mellitus	Yes/No	
	Dyslipidemia	Yes/No	
	Family history of CVD	Yes/No	
	Current medications	List	
F. Thyroid function tests	TSH	mIU/L	
	T3	ng/mL	
	T4	µg/dL	
	Thyroid status	Euthyroid/Hypothyroid/Hyperthyroid	
G. Blood pressure assessment	Systolic BP	mmHg	
	Diastolic BP	mmHg	
	Hypertension status	Normal/Prehypertensive/Hypertensive	
H. ECG findings	Heart rhythm	Normal/Abnormal	
	Arrhythmia	Yes/No	
	Heart rate	bpm	
	Tachycardia	Yes/No	
	Bradycardia	Yes/No	
	Ischemic changes	Yes/No	
	Conduction defect	Yes/No	
	ECG interpretation	Normal/Abnormal	
	ECG reviewer 1	Cardiologist ID	
	ECG reviewer 2	Cardiologist ID	
I. Clinical symptoms	Palpitations	Yes/No	
	Chest pain	Yes/No	
	Dyspnea	Yes/No	
	Fatigue	Yes/No	
J. Final study grouping	Thyroid category	Euthyroid/Hypothyroid/Hyperthyroid	
	Cardiovascular status	Normal/Affected	
